# TLR4‐defective (C3H/HeJ) mice are not protected from cast immobilization‐induced muscle atrophy

**DOI:** 10.14814/phy2.13255

**Published:** 2017-04-21

**Authors:** Noriaki Kawanishi, Risa Nozaki, Hisashi Naito, Shuichi Machida

**Affiliations:** ^1^ Institute of Health & Sports Science and MedicineJuntendo UniversityChibaJapan; ^2^ Research Fellow of the Japan Society for the Promotion of SciencesTokyoJapan; ^3^ Guraduate School of Health and Sports ScienceJuntendo UniversityChibaJapan

**Keywords:** Immobilization, inflammation, muscle atrophy, TLR4

## Abstract

Recent studies have shown that activation of Toll‐like receptor (TLR)4 signaling may be an important factor in muscle atrophy and excessive inflammatory response associated with immobilization. To examine the role of TLR4 signaling on cast immobilization‐induced skeletal muscle atrophy, we tested the hypothesis that muscle atrophy and inflammation after cast immobilization is reduced in TLR4‐defective mice. TLR4‐defective (C3H/HeJ) and wild type (C3H/HeN) mice were divided into control and cast‐immobilization groups. Cast immobilization was imposed for 14 days. Cast immobilization increased TLR4 mRNA expression in the gastrocnemius and decreased muscle mass and cross‐sectional area (CSA) of the gastrocnemius fibers. However, there was no difference in the gastrocnemius muscle mass and CSA between TLR4‐defective and wild type mice. Cast immobilization‐induced increase in ubiquitin E3 ligases (MAFbx/Atrogin‐1 and MuRF1), inflammatory cytokines, and macrophage/monocyte marker mRNAs were unaffected by defective TLR4. Our findings in C3H/HeJ mice suggested that TLR4 signaling might not play an essential role in immobilization‐induced muscle atrophy.

## Introduction

Inactivity due to cast immobilization by injury or fracture, bed rest after treatment, and zero gravity environment in space are well‐known causes of skeletal muscle atrophy. Skeletal muscles are maintained by a balance between synthesis and degradation of muscle proteins, the lack of which causes muscle atrophy (Boonyarom and Inui 2006). Muscle protein degradation plays an important role in skeletal muscle atrophy and in muscle wasting conditions including immobilization and cachexia (Foletta et al. 2011; Okamoto et al. 2011). Pathways of intracellular signal transduction, such as ubiquitin‐proteasome proteolysis, apoptosis, and autophagy, have been associated with muscle protein degradation induced by immobilization and cachexia (Sato et al. 2014).

Recently, excessive inflammatory response has been recognized as a crucial mechanism for muscle atrophy in various models of the disease (Zhang et al. 2007). Inflammatory cytokine [e.g., tumor necrosis factor (TNF)‐*α*, interleukin (IL)‐6, and IL‐1*β*] levels in skeletal muscle of patients with cachexia and septicemia are higher than that in skeletal muscle of healthy individuals, and these cytokines contribute to maintain the pathological chronic inflammatory conditions (Meng and Yu 2010). Moreover, several studies reported that upon immobilization, expressions of inflammatory cytokine genes are increased in atrophied muscles (Caron et al. 2009; Park et al. 2013). Although inflammatory cytokines are released from the immune and parenchyma cells (including muscle cells), they regulate pathways of intracellular signal transduction involved in muscle atrophy (Cohen et al. 2015). In vitro studies have shown that inflammatory cytokines enhance the expression of muscle‐specific ubiquitin ligases such as MAFbx/Atrogin‐1 and MuRF1, which have been linked to the degradation of muscle proteins (Li et al. 2009). Therefore, local inflammation of skeletal muscles induced by immobilization is associated with the development of muscle atrophy. However, the mechanisms underlying muscle atrophy via inflammation induced by immobilization remain to be elucidated.

Secretion of inflammatory cytokines is primarily mediated by transcription factors such as nuclear factor (NF)‐*κ*B and interferon regulatory factor (IRF), and Toll‐like receptors (TLR)4, which are a family of innate cellular pathogen recognition receptors, have been shown to play critical roles in activation of these transcription factors (Takeda and Akira 2004). TLR4 is known to play an essential role in the recognition of microbial components such as lipopolysaccharide (LPS), which is commonly seen on gram‐negative bacteria (Takeuchi et al. 1999). TLR4 also recognizes endogenous ligands such as heat shock protein (HSP) (Erridge 2010). We previously reported that although TLR4 is chiefly expressed on immune cells, it is also expressed on skeletal muscle cells (Kawanishi et al. 2008). Based on the observation that LPS‐induced muscle catabolism and ubiquitin ligases activation was lower in TLR4‐knockout mice than in wild type mice, a recent study hypothesized that TLR4 plays a pathogenic role in muscle atrophy (Doyle et al. 2011). Moreover, Cannon et al. (2007) reported that lean body mass including skeletal muscle were higher and plasma levels of inflammatory cytokines were lower in TLR4‐defective tumor‐bearing mice than in wild type tumor‐bearing mice. Thus, TLR4 signaling mediates muscle atrophy via activation of inflammatory response in cachexia model.

Recently, activation of TLR4 signaling has been considered to be associated with inactivity‐induced muscle atrophy. In fact, reported that TLR4‐knockout mice exhibit decreased mechanical ventilation‐induced diaphragmatic muscle atrophy than that exhibited by wild type mice Schellekens et al. (2012). Interestingly, a recent study showed that even short‐term bed rest can induce increased mRNA levels of inflammatory cytokines and protein levels of TLR4 in the skeletal muscles of healthy older adults (Drummond et al. 2013). Therefore, increased TLR4 expression, by inactivity such as immobilization, may be an important factor in muscle atrophy and excessive inflammatory response. Here, we tested the hypothesis that cast immobilization‐induced muscle atrophy and inflammation is reduced in TLR4‐defective C3H/HeJ mice. The aim of this study is to examine the role of TLR4 signaling on cast immobilization‐induced skeletal muscle atrophy and inflammation.

## Methods

### Animals

Ten‐week‐old male C3H/HeN (*n* = 10) and C3H/HeJ (*n* = 11) mice were purchased from Japan SLC (Shizuoka, Japan). C3H/HeJ mice have a single mutation in TLR4, which makes them unable to respond to LPS and endogenous ligands, and produce inflammatory cytokines (Hoshino et al. 1999; Ohashi et al. 2000). The mice were housed in a controlled environment with a 12‐h light/12‐h dark cycle (lights on at 0900 am). All mice had free access to food and water, and were allowed to acclimatize to their new surroundings for 1 week before the study. The C3H/HeN and C3H/HeJ mice were randomly assigned to two groups: one was untreated (C3H/HeN mice; *n* = 5, C3H/HeJ mice; *n* = 5), while the other was hind limb‐immobilized (C3H/HeN mice; *n* = 5, C3H/HeJ mice; *n* = 6). The experimental procedures followed the Guiding Principles for the Care and Use of Animals of the Juntendo Institutional Animal Care and Use Committee (approval no. H26‐06).

### Hind limb immobilization

Hind limb immobilization of the mice was performed according to a previously described procedure (Cannon et al. 2007). Briefly, the mice were lightly anaesthetized with the inhalant isoflurane (Abbott, Tokyo, Japan) prior to attachment of the casting material. Both the right and left hind limbs of the mice were fixed in a shortened position with casting tape (Scotchcast Plus‐J; 3M Health Care, St. Paul, MN). After casting, the mice were housed one animal per cage and provided free access to standard mouse chow and water. The animals were checked daily for damage to casting material, which was subsequently repaired as necessary. Immobilization was imposed for 14 days. At the end of the immobilization, the casts were removed under isoflurane anesthesia, and skeletal muscles (soleus, plantaris, gastrocnemius, tibialis anterior, and quadriceps femoris) from both the hind limbs were carefully dissected, weighed, and frozen in liquid nitrogen for storage at −80°C.

### Real‐time polymerase chain reaction (qPCR)

Gastrocnemius muscles from the mice were pooled for preparation of the homogenate and for RNA isolation. Total RNA was isolated from the muscle tissues, using TRIzol Reagent (Invitrogen, Carlsbad, CA) according to the manufacturer's protocol. Purity of the total RNA was assessed using the NanoDrop system (NanoDrop Technologies, Wilmington, DE). Total RNA was reverse transcribed to cDNA, using the SuperScript^®^ VILO^™^ cDNA Synthesis Kit (Invitrogen) according to the manufacturer's instructions. PCR was performed in the 7300 real‐time PCR system (Applied Biosystems, Foster City, CA), using the TaqMan Fast Universal PCR Master Mix (Applied Biosystems) and SYBR Green PCR Master Mix (Applied Biosystems). The reaction conditions were as follows: initial denaturation at 95°C for 10 min, followed by 40 cycles of denaturation at 95°C for 15 sec, and annealing at 60°C for 1 min. 18S rRNA was used as the housekeeping gene, and all data are represented as fold change relative to its expression (standard curve method), based on the values for the C3H/HeN untreated group. Primer and probe sets for TLR4 (Mm00445273), MAFbx/Atrogin‐1 (Mm00499518), MuRF1 (Mm01188690), TNF‐*α* (Mm00443258), and IL‐6 (Mm00446190) were purchased from Applied Biosystems. The primer sequences for F4/80 and monocyte chemoattractant protein (MCP)‐1 were as follows: F4/80, 5′‐CTTTGGCTATGGGCTTCCAGTC‐3′ (forward), and 5′‐GCAAGGAGGACAGAGTTTAT‐CGTG‐3′ (reverse); MCP‐1, 5′‐CTTCTGGGCCTGCTGTTCA‐3′ (forward), and 5′‐CCAGCCTAC‐TCATTGGGATCA‐3′ (reverse).

### Muscle fiber cross‐section area (CSA) analysis

Cryostat cross‐sections (10 *μ*m thickness) were cut perpendicular to the left lateral head of gastrocnemius muscles. The frozen sections of the muscle tissues were fixed with 4% paraformaldehyde and incubated in hematoxylin and eosin (H&E) staining solution. CSAs of at least 100 muscle fibers were measured under a fluorescence microscope BZ‐8000, using BZ‐2 software (Keyence, Osaka, Japan).

### Statistical analyses

All data are expressed as mean ± standard error. Statistical analyses were performed using the Statistical Package for the Social Sciences (Version 18.0; SPSS Inc., Chicago, IL). Student *t*‐test was performed to compare data between groups. Tukey hoc tests after one‐way ANOVA were performed for multiple comparisons. The alpha level was set at *P* < 0.05.

## Results

**Figure 1:**
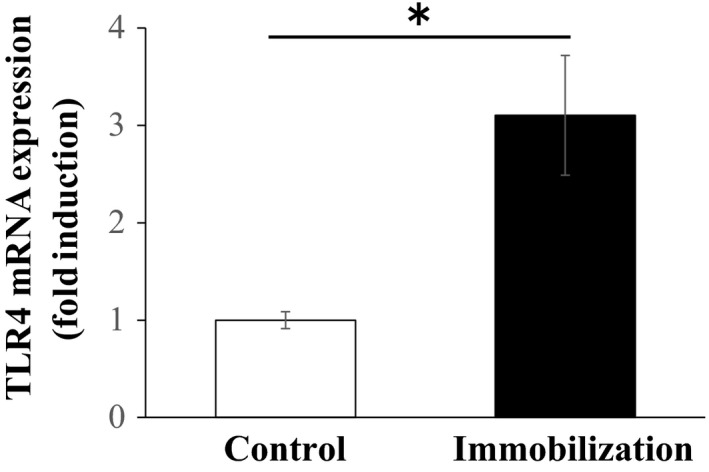
Effect of cast immobilization on Toll‐like receptor (TLR)4 expression levels in C3H/HeN mice (*n* = 5 each group). The results are expressed as mean ± standard error. *P < 0.05.

Since TLR4 is the major factor in muscle atrophy associated with cachexia, we first determined whether cast immobilization increases TLR4 level in the muscles of mice. Indeed, the mRNA levels of TLR4 (3.2‐fold) in the gastrocnemius muscle tissues of immobilized C3H/HeN mice were higher than those in the gastrocnemius muscle tissues of the untreated controls (*P* < 0.05; Fig. 1).

**Figure 2:**
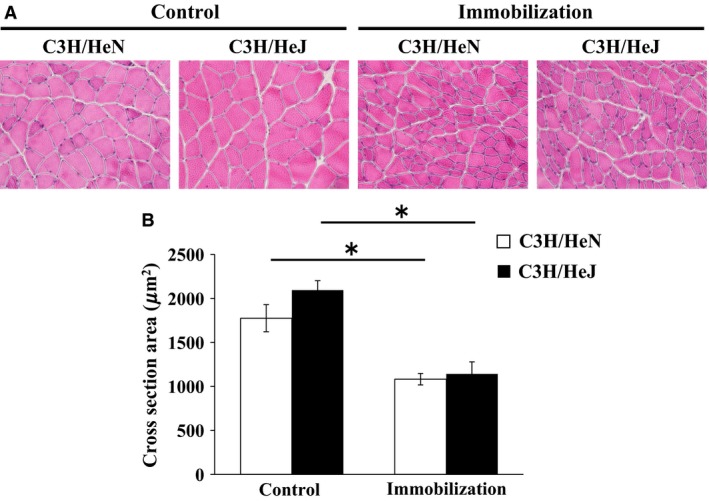
Effect of TLR4 deficiency on muscle atrophy after cast immobilization of mice. (A) Hematoxylin and eosin staining of gastrocnemius muscle sections. (B) Cross‐section area of gastrocnemius muscle fiber in wild type C3H/HeN and nonfunctional‐TLR4 C3H/HeJ mice after 14 days of cast immobilization (*n* = 5–6 each group). The results are expressed as mean ± standard error. *P < 0.05.

We next investigated whether TLR4 mediates cast immobilization‐induced muscle atrophy in TLR4‐defective C3H/HeJ mice. The tissue mass of soleus, plantaris, gastrocnemius, and quadriceps femoris corrected for body weights was lower in the cast‐immobilized C3H/HeN mice than in the untreated C3H/HeN mice (*P* < 0.01 in all the cases). However, there was no difference in the skeletal muscle mass of the two genotypes in the cast‐immobilized mice (gastrocnemius muscle mass change: −16.1 ± 1.7% in C3H/HeN, −15.9 ± 1.7% in C3H/HeJ) on day 14 in the cast‐immobilized mice (Table 1). Furthermore, we performed H&E staining of the skeletal muscle tissues to determine CSAs of the muscle fibers. Our results showed that the CSAs of gastrocnemius muscle fibers in the immobilized mice were smaller than that in the untreated mice. However, there was no difference in the CSAs of muscle fibers in the C3H/HeN and C3H/HeJ mice (Fig. 2).

**Table 1:** The comparison of body mass, soleus, plantaris, gastrocnemius, tibialis anterior, and quadriceps femoris muscle mass between C3H/HeN and C3H/HeJ in cast immobilized and untreatedmice

	Control		Cast immobilization	
	C3H/HeN	C3H/HeJ	C3H/HeN	C3H/HeJ
Body mass (g)				
Initial	27.9 ± 0.6	27.0 ± 0.4	27.9 ± 0.6	27.2 ± 0.6
Final	29.2 ± 0.5	28.2 ± 0.7	24.9 ± 0.4^a^	24.8 ± 0.4^b^
Muscle mass (mg)				
Soleus	7.1 ± 0.3	6.8 ± 0.2	4.4 ± 0.1^a^	4.6 ± 0.1^b^
Plantaris	17.2 ± 0.5	16.4 ± 0.4	11.5 ± 0.3^a^	11.6 ± 0.2^b^
Gastrocnemius	112.9 ± 2.3	107.7 ± 1.3	79.8 ± 1.1^a^	80.0 ± 1.8^b^
Tibialis anterior	58.4 ± 1.3	58.5 ± 1.2	51.4 ± 1.7^a^	52.1 ± 0.8^b^
Quadriceps femoris	175.8 ± 3.6	183.3 ± 1.2	122.2 ± 3.9^a^	125.8 ± 1.6^b^
Muscle mass corrected for body weight (mg/g)				
Gastrocnemius	3.866 ± 0.071	3.820 ± 0.091	3.247 ± 0.053^a^	3.224 ± 0.052^b^
Plantaris	0.590 ± 0.014	0.581 ± 0.011	0.466 ± 0.010^a^	0.471 ± 0.011^b^
Soleus	0.245 ± 0.014	0.239 ± 0.012	0.178 ± 0.004^a^	0.184 ± 0.004^b^
Tibialis anterior	2.001 ± 0.053	2.081 ± 0.078	2.092 ± 0.079	2.106 ± 0.054
Quadriceps femoris	6.008 ± 0.078	6.510 ± 0.198	4.967 ± 0.079^a^	5.076 ± 0.072^b^

Data are presented as mean ± SEM.

a *P *<* *0.05, different from Control C3H/NeH.

b *P *<* *0.05, different from Control C3H/NeJ.

**Figure 3:**
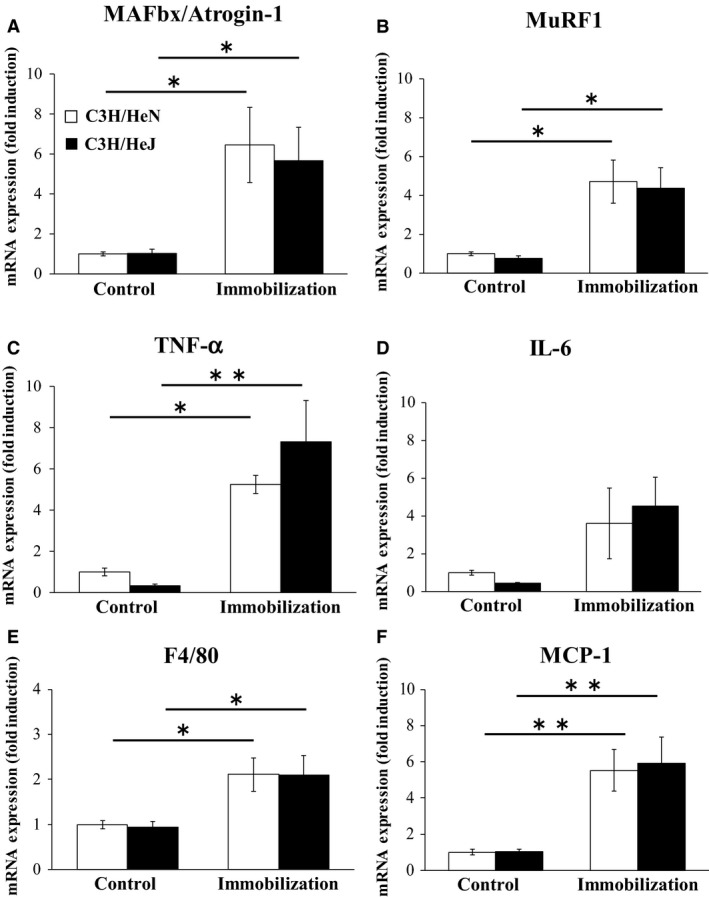
Effect of TLR4 deficiency on protein degradation and inflammation after cast immobilization of mice. mRNA expression of Atrogin1(A), MuRF (B), TNF‐*α* (C), IL‐6 (D), F4/80 (E), and MCP‐1(F) were measured in wild‐type C3H/HeN and nonfunctional‐TLR4 C3H/HeJ mice after 14 days of cast immobilization (*n* = 5–6 each group). The results are expressed as mean ± standard error. **P < 0.01, *P < 0.05.

We next examined the expression of several ubiquitin ligases and inflammatory cytokines in the gastrocnemius muscle tissues. MAFbx/Atrogin‐1 and MuRF1 mRNA levels were significantly higher in the immobilized mice than in the untreated mice (*P* < 0.05). However, there were no differences in these mRNA levels in the C3H/HeN and C3H/HeJ cast‐immobilized mice (Fig. 3A and B). As shown in Figure 3C, the mRNA levels of TNF‐*α* were also affected by cast immobilization (*P* < 0.05). However, this variable did not differ between the cast‐immobilized groups of the two mouse models on day 14 (*P* < 0.05; Fig. 3C). The basal expression levels of TNF‐*α* and IL‐6 in skeletal muscles were lower in the C3H/HeJ mice than in C3H/HeN mice (unpaired *t*‐test, *P* < 0.05; Fig. 3C and D). mRNA levels of F4/80, reflecting the presence of monocytes and macrophages, were significantly higher in the cast‐immobilized mice than in the untreated mice (*P* < 0.01). Similarly, mRNA level of MCP‐1, which is a major macrophage chemokine, also increased following cast immobilization (*P* < 0.05). However, there was no difference in the mRNA levels of F4/80 and MCP‐1 in the cast‐immobilized C3H/HeN and C3H/HeJ mice (Fig. 3E and F).

## Discussion

Cast immobilization can lead to muscle atrophy, which involves loss of fiber size and muscle mass. However, the mechanisms by which cast immobilization reduces muscle mass remain unclear. Although the mechanisms regulating muscle atrophy are complex, activation of TLR4 signaling has been considered to be associated with inactivity‐induced muscle atrophy. In this study, we detected significant elevation in TLR4 mRNA levels in the gastrocnemius muscles of the mice after 14 days of cast immobilization. These results suggest that muscle atrophy by cast immobilization is affected by alteration of TLR4 expression in muscle. Our hypothesis was that cast immobilization‐induced muscle atrophy and inflammation are reduced in TLR4‐defective C3H/HeJ mice than in wild type mice.

In this study, cast immobilization decreased the muscle mass and CSA of muscle fiber after 14 days of cast immobilization. Decrease in CSA in observed in this study (0.55‐fold) was similar to a previous study on cast immobilized‐muscle atrophy model (Min et al. 2011). Importantly, our result indicated a decrease in muscle mass and muscle fiber CSA in both TLR4‐defective C3H/HeJ and wild type mice. Thus, our findings revealed that cast immobilization‐induced muscle atrophy is not affected by the defective TLR4 signaling.

Importantly, a recent study reported that TLR4 knockout mice are protected against hindlimb unloading‐induced muscle atrophy following tail suspension (Kwon et al. 2016). Moreover, Kwon et al. (2015) reported that knockout mice of differentiation primary response (MyD) 88, which mainly regulates TLR4‐mediated inflammatory responses, exhibit decreased hindlimb unloading‐induced muscle inflammation and atrophy compared to wild‐type mice. Discrepancies between the results of our study and those of the previous study (Kwon et al. 2015) may be related to differences in the muscle atrophy model. In addition to reduced mechanical loading, psychological stress and starvation are known to be important factors in immobilization‐induced muscle atrophy (Paul et al. 2012). In fact, cast immobilization has been shown to increase the secretion of stress hormones such as glucocorticoids (Sato et al. 2011), which contribute to induced muscle atrophy by activating the ubiquitin‐proteasome pathway (Schakman et al. 2013). Physiological stress is known to suppress food intake via regulation of appetite hormones (Sominsky and Spencer 2014), and cast immobilization has also been known to reduce food intake (Shibaguchi et al. 2016). In this study, a body weight loss of 17% was observed for cast immobilization for 2 weeks compared to the control group. Importantly, body weight loss was greater after cast immobilization than in the hindlimb unloading model in TLR4 knockout mice (−9.3%) (Kwon et al. 2015). These results indicate that psychological stress is higher in cast immobilization than in hindlimb unloading. Thus, cast immobilization‐induced muscle atrophy may be greatly affected by psychological stress and starvation.

Activation of TLR4 signaling induces inflammatory response, which plays important roles in muscle atrophy induced by cachexia and sepsis associated with local inflammation (Cohen et al. 2015). Recent studies showed increased gene and protein levels of inflammatory cytokines in cast immobilization‐induced atrophied muscles of mice (Park et al. 2013; Kwon et al. 2016). Inflammatory cytokines such as TNF‐*α*, IL‐1*β*, and IL‐6 are known to induce ubiquitin ligases such as MAFbx/Atrogin‐1 and MuRF1, which are related to the degradation of muscle proteins (Kang and Ji 2013). Importantly, it is well known that cast immobilization leads to degradation of proteins in muscles with high expression of MAFbx/Atrogin‐1 and MuRF1 mRNA (Caron et al. 2011; Okamoto et al. 2011; Kang and Ji 2013; Zhu et al. 2013). Interestingly, Caron et al. (2011) reported that suppression of ubiquitin ligases [e.g. MAFbx/Atrogin‐1*,* MuRF1] by proteasome inhibitor treatment attenuates cast immobilization‐induced muscle atrophy in mice. Therefore, this evidence indicates that the activation of local inflammation and ubiquitin‐proteasome pathways by cast immobilization plays a key role in the pathogenesis of muscle atrophy.

In this study, we observed that cast immobilization induced an increase in expression of TNF‐*α* (5.1‐fold). However, this gene expression level was not significantly different in defective‐TLR4 and wild type mice after cast immobilization. Our results also demonstrated that MAFbx/Atrogin‐1 (6.4‐fold) and MuRF1 (4.7‐fold) upregulated following cast immobilization. The increased expression of these genes was similar to previous studies (Bae et al. 2012). However, gene expression levels of ubiquitin ligases were not significantly different in defective‐TLR4 and wild type mice after cast immobilization. This suggests that the activation of local inflammation and ubiquitin‐proteasome pathways by cast immobilization may not require activation of TLR4 signaling. Our results show that muscle atrophy and inflammatory response by cast immobilization are mediated by TLR4 signaling‐independent pathways.

Macrophages are present in the stromal fraction of skeletal muscle, and regulate inflammatory response via production of inflammatory cytokines. Recent evidence indicates that macrophages play key roles in the development of muscle inflammation and pathological conditions (Pillon et al. 2013). Interestingly, Varma et al. (2009) showed that co‐culture with macrophages increased the mRNA levels of Atrogin‐1 and MuRF1 in skeletal muscle cells. Therefore, macrophage infiltration into skeletal muscle may be an important factor in muscle atrophy. Importantly, Zhu et al. (2013) reported that macrophage infiltration is increased in skeletal muscle at day 14 of cast immobilization. We have also found that cast immobilization increases mRNA expression levels of F4/80 (2.1‐fold) and MCP‐1 (5.5‐fold), but this gene expression level was not significantly different in defective‐TLR4 mice and wild type mice after cast immobilization. Therefore, it is possible that the induction of muscle atrophy and inflammatory response in cast immobilized mice was caused by macrophage infiltration. Further studies will be required to determine whether macrophage modulates muscle atrophy and inflammatory response using macrophage depletion model.

In this study, we found that TLR4‐defective mice showed low levels of gene expression of inflammatory cytokines (TNF‐*α*; 0.39‐fold, IL‐6; 0.48‐fold) under normal (non‐pathological) conditions (Fig. 3C and D). In fact, unpaired *t*‐tests showed a difference between cytokine levels in mice with different genotypes. Another study also reported low levels of gene expression of TNF‐*α* and IL‐6 in the liver and lung of TLR4‐defective (C3H/HeJ) mice (Cho et al. 2005; Levy et al. 2006). These data indicate that defective of TLR4 signaling in C3H/HeJ mice might affect the inflammatory state of local tissue in normal condition. Importantly, unlike in the case of inflammatory cytokine expression, our data showed that muscle mass and gene expression of ubiquitin ligases were not significantly different in defective‐TLR4 mice and wild‐type mice under normal conditions (Fig. 3A and B). Thus, differences in TLR4 signaling of two genotypes have little effect on skeletal muscle atrophy in normal young mice. Recently, chronic low‐grade inflammation in skeletal muscle has been recognized as an important causative factor for age‐related muscle wasting (sarcopenia). Therefore, chronic inflammation by modulating TLR4 signaling may be a key factor for development of sarcopenia. Future studies can use TLR4‐defective C3H/HeJ mice as a model to investigate the role of TLR4 signaling on the development of sarcopenia.

## Conclusion

We demonstrated that cast immobilization markedly increases TLR4 mRNA expression in the skeletal muscles of mice. We also evaluated the role of TLR4 signaling in cast immobilization‐induced skeletal muscle atrophy in TLR4‐defective C3H/HeJ mice. However, cast immobilization decreases muscle mass and muscle fiber CSA in both TLR4‐defective and wild type mice. Taken together, our results provide evidence that TLR4 signaling does not play an essential role in cast immobilization‐induced muscle atrophy.

## Conflict of Interest

None declared.
